# Improved Anticancer Properties of Silver Nanoparticles by Albumin Coating in Prostate Cancer Cell Lines: An In Vitro Study

**DOI:** 10.3390/pharmaceutics18030338

**Published:** 2026-03-10

**Authors:** Leila Zareian Baghdadabad, Iman Menbari Oskouie, Seyed Reza Yahyazadeh, Pedram Golmohammadi, Rahil Mashhadi, Mahdi Khoshchehreh, Seyed Mohammad Kazem Aghamir

**Affiliations:** 1 Urology Research Center, Tehran University of Medical Sciences, Tehran 1416753955, Iran; l-zareian@farabi.tums.ac.ir (L.Z.B.); imanmenbary@gmail.com (I.M.O.); pedramgolmohammadi9@gmail.com (P.G.); rh_mashhadi@yahoo.com (R.M.); 2 Department of Urology, Shariati Hospital, Faculty of Medicine, Tehran University of Medical Sciences, Tehran 1419733141, Iran; r.yahyazadeh@gmail.com; 3 Department of Pathology Lab Medicine, David Geffen School of Medicine, University of California, Los Angeles, CA 90095, USA; mkhoshchehreh@mednet.ucla.edu

**Keywords:** albumin-coated silver nanoparticles, prostate cancer, anticancer, gene expression, apoptosis

## Abstract

**Background**: Silver nanoparticles (AgNPs) trigger apoptosis in cancer cells, while albumin nanoparticles enable effective drug delivery. This study compares the antitumor and cytotoxic effects of albumin-coated AgNPs (AgNPs-Alb) versus AgNPs on human prostate cancer cell lines. **Method**: AgNPs-Alb were synthesized and tested against PC3 and LNCaP prostate cancer cell lines. Characterization via Transmission Electron Microscopy (TEM), Dynamic Light Scattering (DLS), and Ultraviolet-Visible (UV-Vis) spectroscopy confirmed their properties. IC50 values were determined using MTT assay, with apoptosis assessed by Annexin-V/PI staining. DNA cell cycle was analyzed by PI staining. Migration, proliferation, and nuclear morphology were evaluated through scratch-wound, colony-forming, and Hoechst staining assays. Gene expression of Snail, E-cadherin, VEGF-C, VEGF-A, Bcl2, Bax, and P53 was analyzed using real-time PCR. **Results**: The IC50 values for AgNPs and AgNPs-Alb were 48 μM and 32 μM in PC3 cells, and 110 μM and 95 μM in LNCaP cells, respectively. AgNPs-Alb significantly inhibited PC3 cell migration compared to AgNPs (*p* < 0.001) and Bicalutamide (*p* < 0.0001). In both cell lines, AgNPs-Alb significantly reduced colony formation compared to AgNPs and Bicalutamide (*p* < 0.05). Flow cytometry revealed a higher percentage of apoptotic cells in PC3 with AgNPs-Alb treatment compared to AgNPs and Bicalutamide. In LNCaP cells, AgNPs-Alb induced a significantly higher percentage of Sub-G1 cells. AgNPs-Alb treatment caused greater mRNA suppression of VEGF-A and a higher Bax/Bcl2 ratio in PC3 and LNCaP cells (*p* < 0.05). Additionally, a significant increase in P53 and E-cadherin, alongside a decrease in VEGF-C expression in LnCAP cells, was observed (*p* < 0.05). **Conclusions**: This study suggests that AgNPs-Alb have stronger anticancer and cytotoxic effects compared to AgNPs alone against PCa cell lines and higher effects were observed on PC3 cells compared to LnCAP cells.

## 1. Introduction

Prostate cancer (PCa) is more common in men and is a major public health problem worldwide, especially in the United States [1]. The GLOBOCAN 2022 reported 1,466,680 newly diagnosed PCa cases, and 396,792 PCa deaths occurred in 2022 across the world [2]. It is also expected to increase to 24 million cases by 2035 [2]. Despite recent advances in therapeutic modalities such as chemotherapy, radiotherapy, and radical prostatectomy, which can control localized PCa, metastatic PCa remains challenging [3,4]. In addition, the use of radiotherapy and standard chemotherapeutic drugs produces pronounced and significant side effects in patients [5]. There is a critical need for novel approaches in PCa therapy to enhance treatment effectiveness while minimizing adverse effects. Nanoparticles (NPs) have emerged as promising platforms in this field, with silver nanoparticles (AgNPs) increasingly being explored for their antitumor properties. Recent studies have demonstrated encouraging outcomes for AgNPs in cancer management [6]. These nanoparticles are capable of cellular entry via endocytosis, where they can disrupt chromosomal stability within the nucleus and impair the process of mitosis [7]. Several molecular pathways are targets of AgNPs, including angiogenesis, apoptosis, and epithelial–mesenchymal transition (EMT) [8,9,10]. The efficacy of AgNPs can be improved under store release conditions, which need to be coated by some components to improve the lifetime treatment impact and reduce toxicity [11]. Developing a successful chemotherapeutic agent requires targeting cancer cells while minimizing harm to healthy tissues, particularly through controlled release mechanisms.

Previous studies have demonstrated that silver nanoparticles exhibit strong anticancer activity against multiple cancers, including prostate cancer [12,13,14,15]. However, the clinical application of silver nanoparticles has been hindered by toxic and genotoxic effects observed at elevated doses [16]. Consequently, developing strategies like nanoformulation to specifically deliver silver nanoparticles to cancer cells while minimizing toxicity to normal cells remains a vital area of investigation. Albumin is an essential plasma protein that regulates blood osmotic pressure and carries a variety of substances to cells under normal physiological conditions. This macromolecule has been extensively utilized as a coating agent because of its outstanding properties, such as high water solubility, low toxicity, excellent biocompatibility, prolonged blood half-life, minimal immunogenicity, ease of purification, and targeted accumulation within the tumor microenvironment [17,18]. Albumin possesses unique properties that make it an ideal candidate for nanoformulation. The presence of carboxyl and amino functional groups in albumin enables its interaction with diverse molecules [19]. Albumin also assists in endocytosis and transcytosis by interacting with proteins such as albondin/glycoprotein 60 [20]. These characteristics enable its effective delivery of anticancer drugs and agents against various cancer types, showing promising therapeutic outcomes. For instance, Solanski et al. demonstrated that the antitumor activity and cellular uptake of silibinin in cervical cancer cells were significantly increased when delivered via albumin nanoparticles [21]. However, the influence of albumin silver nanoparticles on prostate cancer cells remains unexplored so far.

In this study, the anticancer properties of albumin-coated AgNPs (AgNPs-Alb) were evaluated in comparison to AgNPs against metastatic human PCa cell lines.

## 2. Materials and Methods

### 2.1. AgNPs Synthesis and Preparation

The materials employed in the experiments included silver nitrate (AgNO_3_, CAS: 7761-88-8), Polyvinylpyrrolidone, (PVP, CAS: 9003-39-8), and bovine serum albumin (BSA, CAS: 9048-46-8). These chemicals were obtained from Sigma Aldrich, St. Louis, MO, USA, and were utilized in their original form without any modifications. AgNPs were synthesized using polyvinylpyrrolidone (PVP) as the principal reagent (facile synthesis of PVP-coated silver nanoparticles and evaluation of their physicochemical, antimicrobial and toxic activity) [22]. A 2% solution of PVP was prepared by dissolving 5 g of PVP in 250 mL of deionized water under stirring at room temperature. Separately, 0.1 g of silver nitrate (AgNO_3_) was dissolved in 0.5 mL of deionized water and then added dropwise to 25 mL of the 2% PVP solution with continuous stirring. This resulted in a final AgNO_3_ concentration of approximately 3.92 mg/mL. The mixture was heated at 100 °C for one h in the dark, leading to the formation of pale brown silver nanoparticles (AgNPs). For the synthesis of the AgNPs-BSA (AgNPs-Alb), bovine serum albumin (BSA) and AgNPs were combined in a solution of phosphate-buffered saline (PBS) and dichloromethane at a volume ratio of 99:1. The mixture was adjusted to final concentrations of 5% (*w*/*v*) BSA and 1 mM AgNPs. Subsequently, 20 µL of ethanol and 230 µL of dichloromethane were added, and the solution was gently stirred for 5 min to form a coarse emulsion. The emulsion was then subjected to high-pressure homogenization using a Bandelin Sonopuls ultrasonic homogenizer (Bandelin electronic GmbH & Co. KG, Berlin, Germany) at 20 kHz and room temperature until a milky suspension was obtained. The suspension was transferred to a rotary evaporator where dichloromethane was removed at 40 °C under a reduced pressure of 30 mm Hg over 20–30 min.

### 2.2. AgNPs Characterization by TEM, Ultraviolet-Visible, and DLS

AgNPs and AgNPs-Alb underwent characterization by transmission electron microscopy (TEM; Zeiss-EM10 C, Carl Zeiss Microscopy GmbH, Oberkochen, Germany) to assess their size and shape, using an accelerating voltage of 80 kV. Their optical properties were explored by measuring absorption spectra using an ultraviolet-visible (UV-vis) spectrophotometer (Model M501, CamSpec Ltd., Leeds, UK) at a 2 nm resolution in the 200–1000 nm wavelength spectrum. Additionally, the sizing of AgNPs was evaluated through Dynamic Light Scattering (DLS; Particle Sizing Systems, Port Richey, FL, USA) employing the NICOMP 380/ZLS (PSS) instrument (Particle Sizing Systems, Port Richey, FL, USA) and the data was processed using ZPW388 software (V2.17.0215, Particle Sizing Systems, Port Richey, FL, USA).

### 2.3. Cell Lines

The LNCaP (ATCC: CRL-10995; NCBI: C439) and PC3 (ATCC: CRL-1435; NCBI: C427) prostate cancer cell lines were obtained from the National Cell Bank at the Pasteur Institute, Tehran, Iran. The cells were cultured in Dulbecco’s Modified Eagle Medium (DMEM; Gibco, Carlsbad, CA, USA), which was supplemented with 10% heat-inactivated fetal bovine serum (FBS), 100 U/mL penicillin, 2 mM L-glutamine, and 100 µg/mL streptomycin (Gibco BRL, Grand Island, NY, USA). Cultures were maintained at 37 °C in a humidified incubator with 5% CO_2_. For controls, cells were exposed to complete DMEM containing either 1% dimethyl sulfoxide (DMSO; Sigma-Aldrich, St. Louis, MO, USA) or phosphate-buffered saline (PBS). Bicalutamide (32.5 mM), used for cell treatments, was dissolved in DMSO as a stock solution.

### 2.4. Analysis of Cell Survival

The MTT assay was employed to assess the suppressive impact of Bicalutamide, AgNPs, and Alb-AgNPs on the metabolic activity of PC3 and LNCaP cell lines. These cells were seeded at a density of 5000 cells per well in 96-well plates and allowed to incubate for 24 h. The experiment introduced varying concentrations of Bicalutamide, AgNPs, and AgNPs-Alb administered separately. The incubation was carried out at 37 °C in a 5% CO_2_ environment. To prepare the MTT solution, 5 mg of 3-[4,5-dimethylthiazol-2-yl]-2,5-diphenyltetrazolium bromide powder (Sigma-Aldrich, St. Louis, MO, USA) was dissolved in 1 mL of sterile PBS, making a 5 mg/mL solution. This was then diluted to a final volume of 10 mL with sterile PBS, yielding a 0.5 mg/mL concentration, which should be freshly prepared for each experiment. After 24 h of exposure to the treatments, wells received 100 μL of the 0.5 mg/mL MTT solution, followed by a 4 h incubation at 37 °C for metabolic activity realization. Formazan products were solubilized with 100 µL of DMSO, producing a purple solution. An ELISA microplate reader (MPR4+, Hiperion, Medizintechnik GmbH & Co. KG, Dorsten, Germany) measured the absorbance at 545 nm.

To identify the half-maximum inhibitory concentration (IC50) values for AgNPs, AgNPs-Alb, and Bicalutamide against both PC3 and LNCaP cells, experiments were repeated in triplicate. Dose–response curves were generated and analyzed using the GraphPad PRISM version 9 software (San Diego, CA, USA) to ascertain the IC50, reflecting the concentration at which cell growth is halved in comparison to the control. The formula applied to calculate the percentage of cell viability from the MTT assay results is as follows:

(1) $$\frac{\left(average\;absorbance\;of\;triplicate\;drug\;wells\right)}{average\;absorbance\;of\;control\;wells}\times 100\%=\%\;cell\;viability$$

### 2.5. Characterization of Cell Morphology

PC3 and LNCaP cells were seeded at a density of 5 × 10^4^ cells per well in six-well plates and treated for 24 h with AgNPs, AgNPs plus Bicalutamide, or Bicalutamide alone. After treatment, cells were washed twice with PBS and fixed with 4% paraformaldehyde. The fixed cells were subsequently examined under an inverted microscope.

### 2.6. In Vitro Colony Formation Assay

Colony formation assay was used to evaluate the invasive capacity of prostate cancer cells in culture. PC3 and LNCaP cells were plated at a density of 1 × 10^3^ cells per well in six-well plates and incubated at 37°C until colonies became detectable by microscopy. Cells were then exposed to AgNPs, AgNPs-Alb, or Bicalutamide for 24 h. After ten days, cultures were washed twice with PBS and fixed with 4% paraformaldehyde. Subsequently, cells were stained with 0.5% crystal violet (*w*/*v*) for 20 min at room temperature. Images were acquired from each well, and colony numbers were quantified using ImageJ software, version 1.54g.

### 2.7. Staining of Cells with Hoechst Dye (33342)

Apoptosis in cancer cells was assessed by Hoechst staining. LNCaP and PC3 cells were seeded in 96-well plates at a density of 2 × 10^3^ cells per well and treated with AgNPs, AgNPs-Alb, or Bicalutamide for 24 h. Following treatment, cells were fixed with 100 μL of cold 4% paraformaldehyde for 20 min and washed twice with PBS. Each well then received 2 μL of Hoechst dye and was incubated in the dark at room temperature for 20 min. Nuclear condensation and fragmentation, indicative of apoptosis, were evaluated by fluorescence microscopy (OPTIKA S.r.l., Ponteranica, Bergamo, Italy) at 100× magnification.

### 2.8. Measurement of Cell Migration

PC3 and LNCaP cells were cultured in six-well plates at a density of 5 × 10^5^ cells per well. Upon reaching approximately 85% confluence, a linear scratch was made in the center of each well using a sterile pipette tip. The wells were rinsed with PBS to remove detached cells. Control wells received only PBS, while other wells were treated with AgNPs, AgNPs-Alb, or Bicalutamide. Images were acquired at 0 and 24 h to document wound closure. The captured images were analyzed, and wound healing rates were quantified using ImageJ software, version 1.54g, according to the following formula:

(2) $$1-\frac{area\;of\;wound\;at\;day\;1}{area\;of\;wound\;at\;day\;0}\times 100\%=\%\;wound\;closure$$

### 2.9. Measurement of Cell Apoptosis by Flow Cytometry

Cell apoptosis assessment was carried out following the protocol provided with the Fluorescein-conjugated annexin V (annexin V-FITC) staining kit. LNCAP and PC3 cells were grown in six-well plates, with each well containing 5 × 10^5^ cells, and exposed to AgNPs, AgNPs-Alb, and Bicalutamide for 24 h. After this period, the cells were rinsed twice with PBS and stained with 100 μL of an annexin-V and Propidium Iodide (PI) blend for 15 min at room temperature in the dark. The fluorescence of the stained cells was then measured using flow cytometry. The apoptosis rate was estimated by determining the proportion of cells that were annexin V+/PI−, employing a BD flow cytometer for this purpose. The analysis was executed using FlowJo software (Tree Star Inc., version 9.6.3, Ashland, OR, USA).

### 2.10. DNA Cell Cycle Analysis

The cell cycle analysis was performed via PI staining method. Both PC3 and LNCaP cells were cultured at a density of 5 × 10^5^ cells per well and subjected to AgNPs, AgNPs-Alb, and Bicalutamide for 24 h. Following this incubation, the cells were washed twice with PBS, fixed in 70% ethanol at −20°C overnight, and washed again. Then, they were treated with RNase I (100 μg/mL) and stained with 500 μL of PI (50 μg/mL in 0.1% Triton X−100/0.1% sodium citrate) for 30 min at a temperature of 37°C. A BD flow cytometer was utilized to categorize the cells, and flow cytometry analyzed the DNA content, specifically identifying apoptotic cells through their sub-G0/G1 hypodiploid DNA content. The Flowjo software (Tree Star Inc., version 9.6.3, USA) was employed for data analysis.

### 2.11. RNA Extraction, cDNA Synthesis, and Gene Expression Analysis by Real-Time PCR

Adhering to the manufacturer’s protocol for RiboEx^TM^, total RNA was isolated. Briefly, RNA was extracted cells using RiboEx^TM^ reagent. After cell disruption and incubation, the mixture was centrifuged, and the supernatant was collected. Chloroform was added, followed by vigorous shaking and another centrifugation step, after which the aqueous phase was recovered. RNA was then precipitated by adding isopropanol and centrifuging. The resulting RNA pellet was washed with 75% ethanol and centrifuged again to finalize the RNA extraction. The RNA purity and concentration were quantified using a Nanodrop ND-1000 spectrophotometer (Nanodrop Technologies, Wilmington, DE) at absorbances of 260 and 280 nm. Subsequently, four micrograms of the total RNA were reverse transcribed into complementary DNA (cDNA) using the PrimeScript RT reagent Kit (Pars Toos. Co., Mashhad, Iran, CAT#: A101161). Following standardization, the cDNAs underwent amplification in a real-time PCR cycler from QIAGEN. The normalization of gene expression levels utilized the B2M housekeeping gene. Relative gene expression calculations were performed using the 2^−∆∆CT^ method. The primers employed and the specific lengths of their amplicons are detailed in Table 1.

### 2.12. Statistical Analysis

The information, presented as average ± standard error from three independent experiments, underwent statistical examination via ANOVA and *t*-tests. The results were considered statistically significant with * *p* < 0.05, ** *p* < 0.01, *** *p* < 0.001, and **** *p* < 0.0001 in comparison to control groups.

## 3. Results

### 3.1. Characterization of AgNPs and AgNPs-Alb

**Figure 1:**
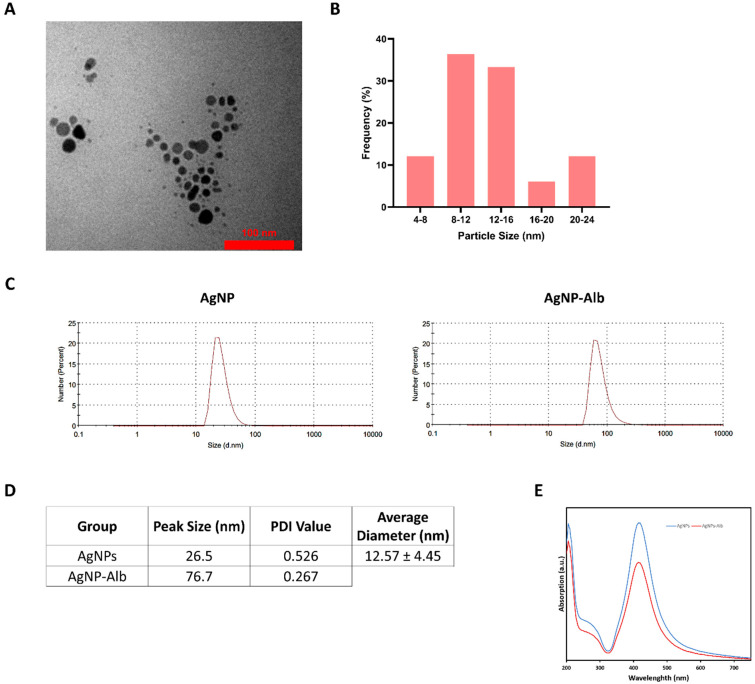
(**A**) TEM images indicating the spherical shape of AgNPs. (**B**). Size diagram of TEM images. (**C**) DLS spectrum of AgNPs and AgNPs-Alb. (**D**) Peak size, PDI value of DLS analysis, and average diameter of AgNPs using TEM. (**E**) UV-Vis’s spectrum of AgNPs and AgNPs-Alb indicating maximum peak at 414.5 nm.

AgNPs was successfully synthesized according to our protocol using PVP agent. PVP was used in our synthesis due to its dual role as a strong stabilizer and reducing agent. Using fewer reagents in synthesis results in decreased side reactions, fewer production stages, and faster manufacturing with lower expenses. The morphology of AgNPs was characterized by TEM, revealing predominantly spherical particles with a range of diameters, as shown in Figure 1A,B. TEM analysis determined that the mean diameter of AgNPs was 12.57 ± 4.45 nm. The particles appeared as monodispersed dark spots with a uniform structure. DLS measurements (Figure 1C,D) showed AgNPs had a particle size distribution from 15.69 to 68.06 nm, with an average diameter of 26.5 ± 8.88 nm. In contrast, AgNPs-Alb particles ranged from 43.82 to 141.8 nm, with a mean diameter of 76.70 ± 30.9 nm. DLS typically reports larger sizes than TEM because it measures the hydrodynamic diameter. Moreover, the fixation and dehydration steps before TEM significantly affect the actual particle sizes. An ultraviolet-visible spectrum analysis was also performed for AgNPs and AgNPs-Alb, and the spectrum revealed a clear absorption peak at 414.5 nm for both structures (Figure 1E). The maximum absorption wavelength of silver nanoparticles in the UV-Vis spectrum typically falls within the range of 400 to 450 nanometers (nm) [23,24]. This absorption is primarily due to the surface plasmon resonance (SPR) phenomenon, which is a collective oscillation of the conduction electrons at the surface of the nanoparticles when they are excited by light.

### 3.2. The Effect of AgNPs, AgNPs-Alb, and Bicalutamide on Cell Proliferation

**Figure 2:**
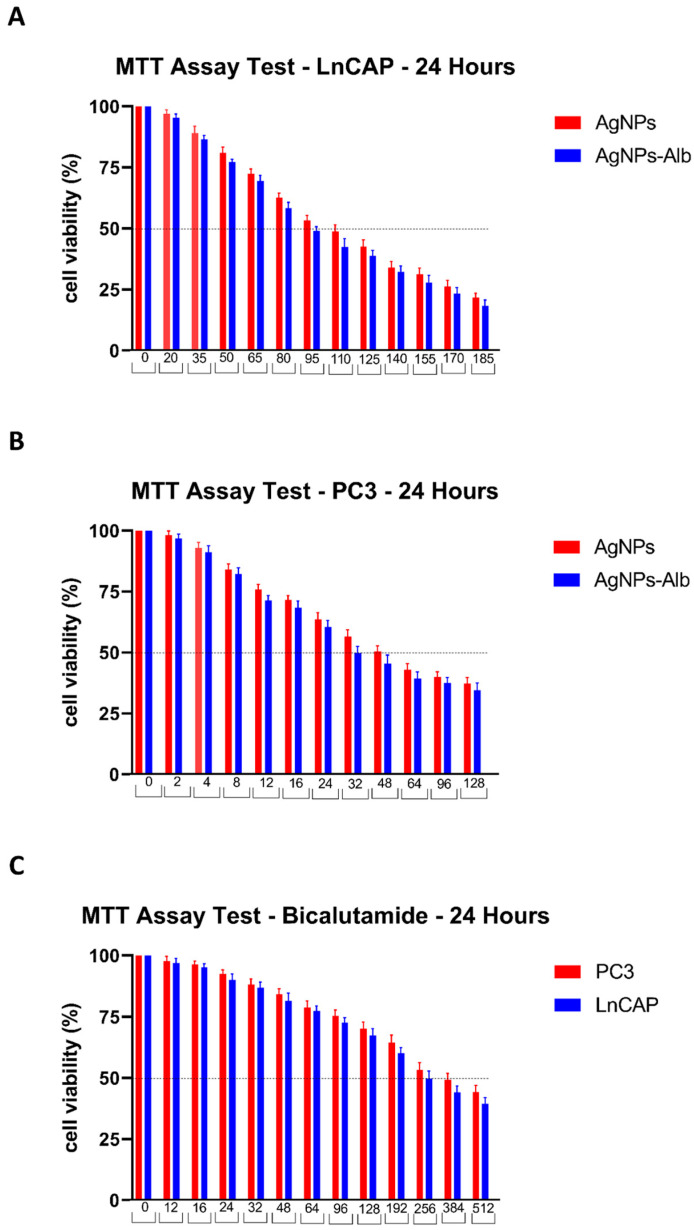
Results of MTT assay test after treating LNCaP and PC3 cell lines with various concentrations of AgNPs, AgNPs-Alb, and Bicalutamide.

The cytotoxic effects of AgNPs, AgNPs-Alb, and Bicalutamide on PC3 and LNCaP cell lines were evaluated. As illustrated in Figure 2, the 24 h IC_50_ values for AgNPs and AgNPs-Alb in PC3 cells were 48 ± 0.32 μM and 32 ± 0.08 μM, respectively, while in LNCaP cells the values were 110 ± 1.74 μM and 95 ± 1.32 μM, respectively. For Bicalutamide, the IC_50_ values after 24 h were 384 ± 4.36 μM for PC3 and 256 ± 0.88 μM for LNCaP. These findings demonstrate that AgNPs, AgNPs-Alb, and bicalutamide induce significant, dose-dependent cytotoxicity in both cell lines. The IC_50_ values were summarized in Table 2. Subsequent experiments utilized concentrations corresponding to half the IC_50_ for each agent over a 24 h period: 24 μM AgNPs, 16 μM AgNPs-Alb, and 192 μM Bicalutamide for PC3 cells; and 55 μM AgNPs, 48 μM AgNPs-Alb, and 124 μM Bicalutamide for LNCaP cells.

### 3.3. Morphological Changes

**Figure 3:**
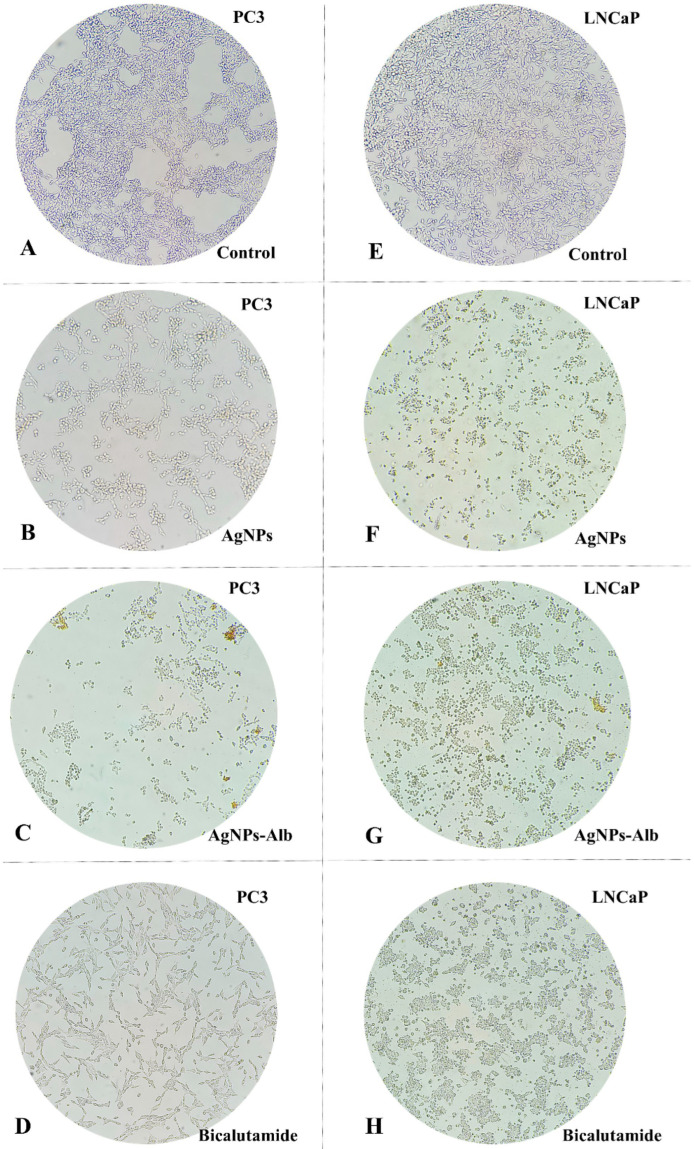
Morphological changes in prostate cancer cells line after treatment with AgNPs, AgNPs-Alb, and Bicalutamide. (**A**) PC3—control group, (**B**) PC3 treated with AgNPs, (**C**) PC3 treated with AgNPs-Alb, (**D**) PC3 treated with Bicalutamide, (**E**) LNCaP—control group, (**F**) LNCaP treated with AgNPs, (**G**) LNCaP treated with AgNPs-Alb, (**H**) LNCaP treated with Bicalutamide.

Assessing cellular morphology plays a vital role in understanding cell behavior. In this study, PC3 and LNCaP cell lines were exposed to AgNPs, AgNPs-Alb, and Bicalutamide for 24 h, after which morphological changes were evaluated using an inverted microscope. As shown in Figure 3, cells treated with AgNPs, AgNPs-Alb, and Bicalutamide exhibited noticeable morphological changes, including cell shrinkage, rounding, elongation, formation of membrane protrusions, and a reduction in viable cell count compared to the control group. These alterations suggest that the tested agents may induce apoptosis in both PC3 and LNCaP cells. Notably, there were no significant differences in the observed cellular morphology between the AgNPs and AgNPs-Alb treatment groups (Figure 3).

### 3.4. Effects of AgNPs, AgNPs-Alb, and Bicalutamide on the Nuclei of PCa Cell Lines

**Figure 4:**
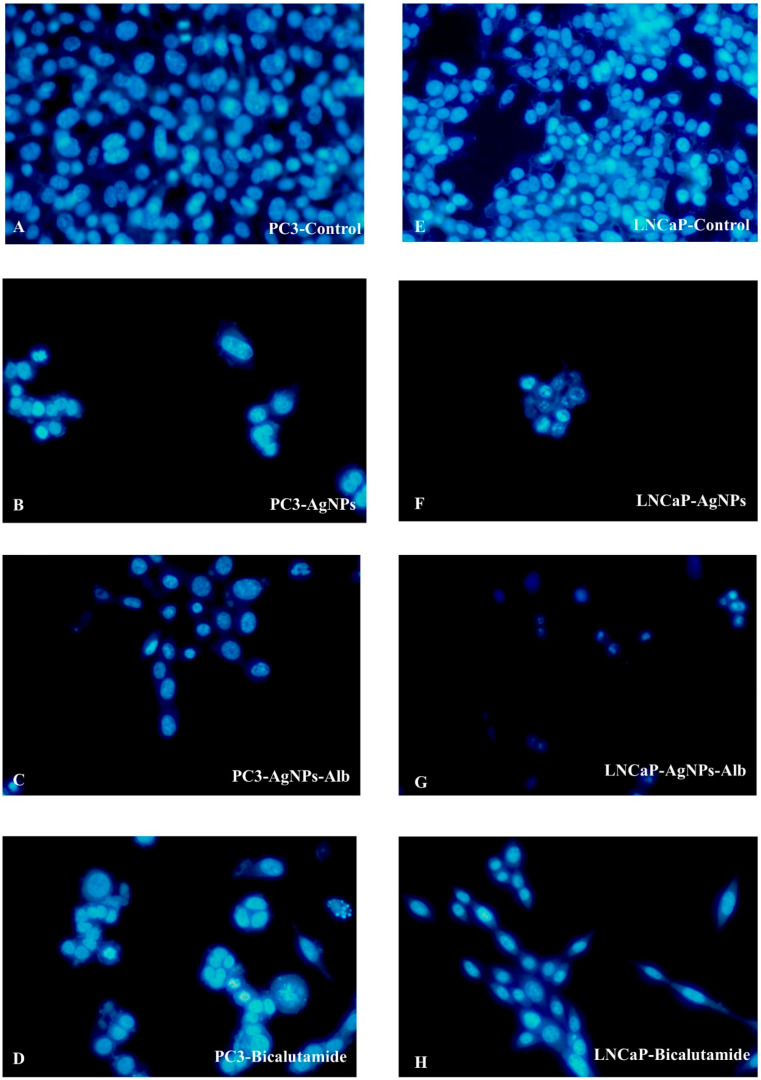
Hoechst dye (33342) fluorescent staining of the prostate cancer cell lines following treatment with AgNPs, AgNPs-Alb, and their combinations. (**A**) PC3—control group, (**B**) PC3 treated with AgNPs, (**C**) PC3 treated with AgNPs-Alb, (**D**) PC3 treated with Bicalutamide, (**E**) LNCaP—control group, (**F**) LNCaP treated with AgNPs, (**G**) LNCaP treated with AgNPs-Alb, (**H**) LNCaP treated with Bicalutamide.

To assess apoptosis, PC3 and LNCaP cell lines were treated with AgNPs, AgNPs-Alb, and Bicalutamide for 24 h, followed by evaluation using fluorescent microscopy and Hoechst staining. As illustrated in Figure 4, Hoechst 33342 staining revealed that nuclei in the control group emitted uniform blue fluorescence. In contrast, cells exposed to AgNPs, AgNPs-Alb, and Bicalutamide displayed marked changes in nuclear morphology, including nuclear fragmentation and disintegration, leading to scattered nuclear material after 24 h. These observations, captured under a fluorescent microscope, further demonstrated features consistent with apoptosis in the treated groups (Figure 4).

### 3.5. Effects of AgNPs, AgNPs-Alb, and Bicalutamide on the Migration of the Prostate Cancer Cells

**Figure 5:**
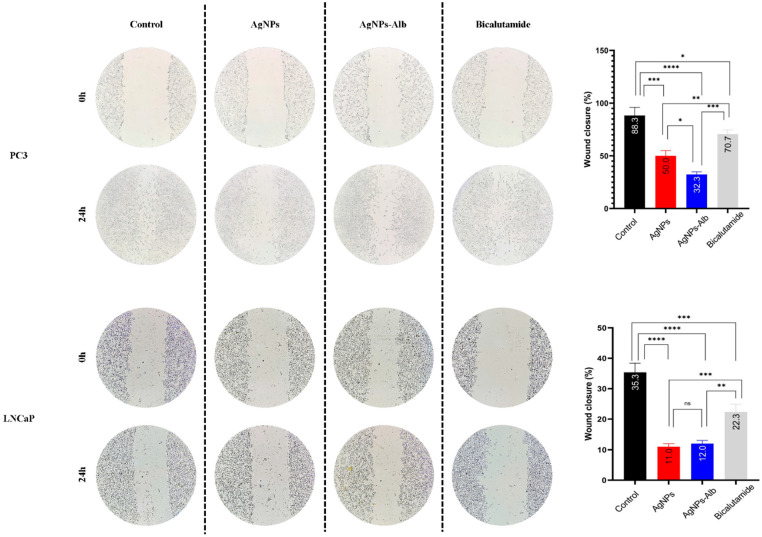
Examination of migration in PC3 and LNCaP cells. A significant restriction in the migration of PC3 and LNCaP cells into the wound area was observed in all treatment groups after 24 h. Statistical significance thresholds were set at * *p* < 0.05, ** *p* < 0.01, *** *p* < 0.001, and **** *p* < 0.0001. ns: no significant difference.

**Figure 6:**
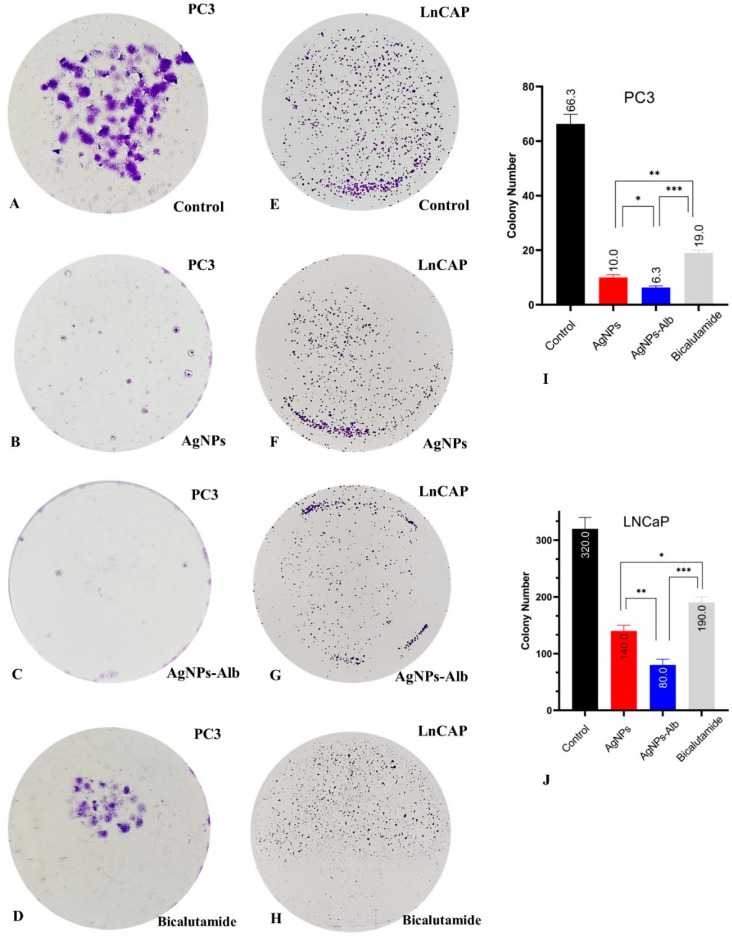
Colony formation assay in PC3 (**A**–**D**) and LNCaP (**E**–**H**) prostate cancer cells. (**I**,**J**): colony number of control and each treated group. Statistical significance thresholds were set at * *p* < 0.05, ** *p* < 0.01, and *** *p* < 0.001.

As shown in Figure 5, treatment with AgNPs, AgNPs-Alb, and Bicalutamide for 24 h led to a significant reduction in the migratory capacity of prostate cancer cells compared to the control group. The wound closure percentages for both treated and control conditions in all cell lines are presented in Figure 5 and Figure 6. In the control group, the cell-free gap was almost completely filled after 24 h. Notably, AgNPs-Alb markedly suppressed migration in PC3 cells relative to both the AgNPs and Bicalutamide treatments (*p* < 0.001 and *p* < 0.0001, respectively). However, in the LNCaP cell line, there was no statistically significant difference in migration inhibition between the AgNPs and AgNPs-Alb groups (*p* > 0.05).

### 3.6. Decrease in the Number of Colonies by AgNPs, AgNPs-Alb, and Bicalutamide

There was a distinct association between the metastatic potential of the prostate cancer cell lines and their ability to form colonies in the absence of treatment. The untreated PC3 cells demonstrated a greater number of colonies than the LNCaP cell line. Treatment with AgNPs, AgNPs-Alb, and Bicalutamide significantly reduced colony formation in both cell lines when compared to the untreated controls (Figure 6). Furthermore, AgNPs-Alb treatment led to a more pronounced suppression of colony formation than either AgNPs or Bicalutamide alone in both cell lines (*p* < 0.05).

### 3.7. Induction of Apoptosis by AgNPs, AgNPs-Alb, and Bicalutamide

Flow cytometry analysis was performed to assess the induction of apoptosis by AgNPs, AgNPs-Alb, and Bicalutamide. In the absence of treatment, the PC3 cell line exhibited baseline early and late apoptosis rates of 1.90% and 1.66%, respectively, while the LNCaP cell line showed rates of 4.05% and 0.46%. Following exposure to AgNPs, these values increased markedly to 20.9% (early) and 26.2% (late) in PC3 cells, and 70.7% (early) and 2.25% (late) in LNCaP cells. Treatment with AgNPs-Alb resulted in early and late apoptosis rates of 44.4% and 21.4% in PC3 cells, and 66.7% and 3.86% in LNCaP cells. When treated with Bicalutamide, apoptosis in PC3 cells was 11.5% (early) and 20.9% (late), while in LNCaP cells the respective values were 50.3% and 7.59%.

**Figure 7:**
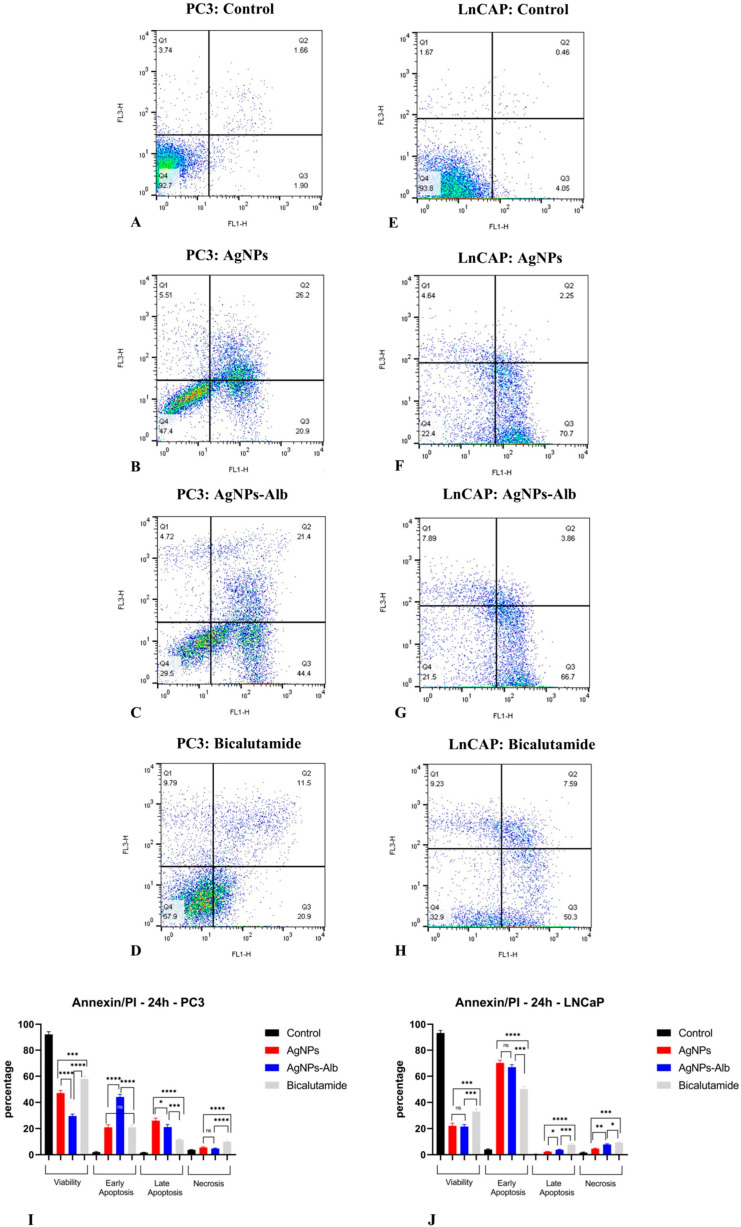
Analysis of PC3 and LNCaP cell apoptosis at 24 h by flow cytometry using Annexin-V-Flous. (**A**–**D**) The apoptosis induction in PC3 cells untreated and treated with AgNPs, AgNPs-Alb, and Bicalutamide. (**E**–**H**) The apoptosis induction in LNCaP cells untreated and treated with AgNPs, AgNPs-Alb, and Bicalutamide. (**I**,**J**) A notable increase in apoptosis rates was observed in all treated groups compared to the untreated control in PC3 and LNCaP cell lines. Statistical significance thresholds were set at * *p* < 0.05, ** *p* < 0.01, *** *p* < 0.001, and **** *p* < 0.0001. ns: no significant difference.

In the PC3 cell line, the percentage of early apoptotic cells in the AgNPs-Alb group was significantly higher than in the AgNPs group, but this difference was not significant in the LNCaP cell line. Additionally, in both the PC3 and LNCaP cell lines, the percentage of necrotic cells was significantly greater in the group of cells that had been administered Bicalutamide (Figure 7).

### 3.8. AgNPs, AgNPs-Alb, and Bicalutamide Induce SubG1-G1 Arrest in the Prostate Cancer Cell Lines

**Figure 8:**
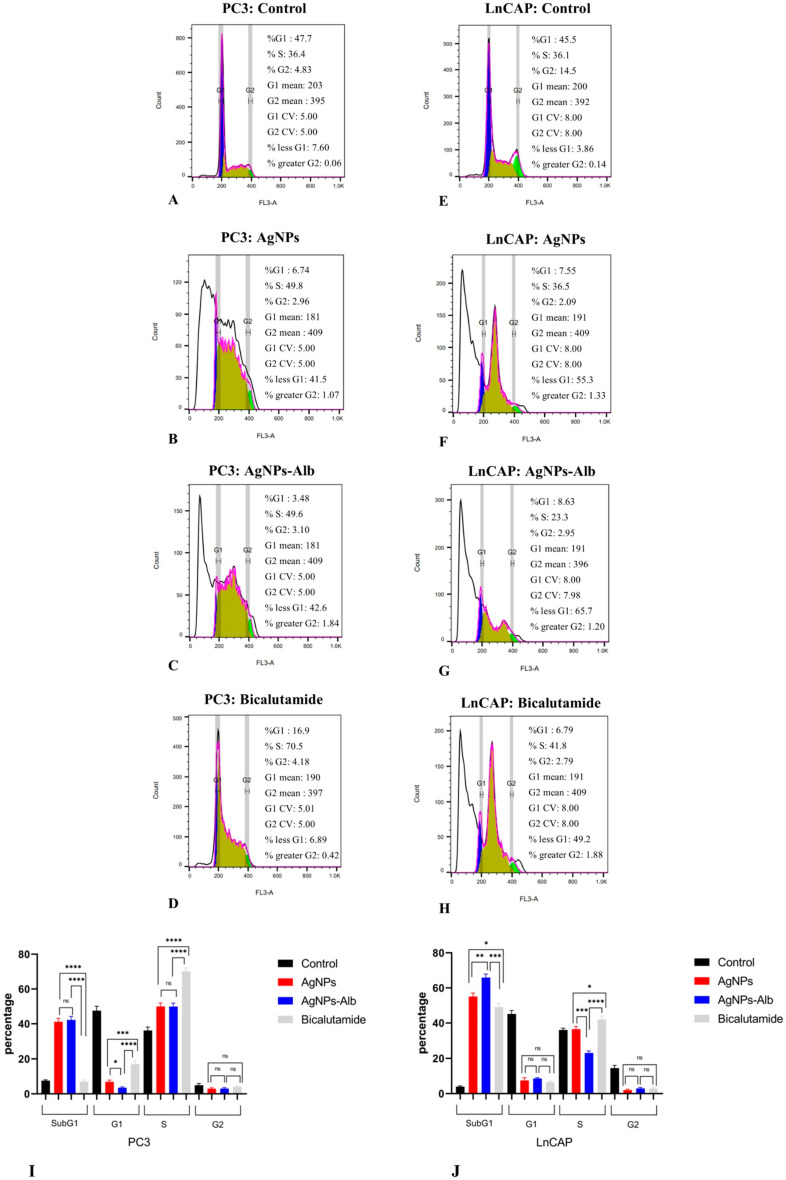
Analysis of the cell cycle in PC3 and LNCaP cell lines. (**A**–**D**) Examination of the cell cycle in untreated PC3 cells and those treated with AgNPs, AgNPs-Alb, and Bicalutamide. (**E**–**H**) Examination of the cell cycle in untreated LNCaP cells and those treated with AgNPs, AgNPs-Alb, and Bicalutamide (**I**,**J**) Cell cycle analysis revealed a significant increase in the sub-G1 phase in PC3 and LNCaP cells treated with AgNPs, AgNPs-Alb, and Bicalutamide. Statistical significance thresholds were set at * *p* < 0.05, ** *p* < 0.01, *** *p* < 0.001, and **** *p* < 0.0001. ns: no significant difference.

Figure 8 presents the flow cytometry analysis of cell cycle distribution in prostate cancer cell lines following treatment with AgNPs, AgNPs-Alb, and Bicalutamide. In PC3 cells, exposure to AgNPs resulted in a significant elevation in the proportion of sub-G1 cells (from 7.6% to 41.5%, *p* < 0.0001) and S phase cells (from 36.4% to 49.8%, *p* < 0.01), accompanied by a marked reduction in G1 (from 47.7% to 6.74%, *p* < 0.0001) and G2/M phase cells (from 4.83% to 2.96%, *p* < 0.05). Similarly, AgNPs-Alb treatment led to increases in sub-G1 (from 7.6% to 42.6%, *p* < 0.0001) and S phase cells (from 36.4% to 49.6%, *p* < 0.05), with corresponding decreases in G1 (from 47.7% to 3.48%, *p* < 0.0001) and G2/M phases (from 4.83% to 3.1%, *p* < 0.05). Bicalutamide administration significantly elevated the S phase population (from 36.4% to 70.5%, *p* < 0.0001) while reducing the percentage of cells in G1 (from 47.7% to 16.9%, *p* < 0.0001).

Similar results were observed in LNCaP prostate cancer cells, showing an increase in sub-G1 phase cells following treatment with AgNPs (3.86% to 55.3%, *p* < 0.0001), AgNPs-Alb (3.86% to 65.7%, *p* < 0.0001), and Bicalutamide (3.86% to 49.2%, *p* < 0.0001).

In the PC3 cell line, there was no significant difference in Sub-G1 cells among AgNPs and AgNPs-Alb (*p* > 0.05), but both had a greater population of Sub-G1 cells compared to bicalutamide (*p* < 0.0001). In the LNCaP cell line, AgNPs-Alb had a significantly greater number of Sub-G1 cells compared to the other treated groups (Figure 8).

### 3.9. Effect of AgNPs, AgNPs-Alb, and Bicalutamide on Gene Expression Levels in Prostate Cancer Cell Line

**Figure 9:**
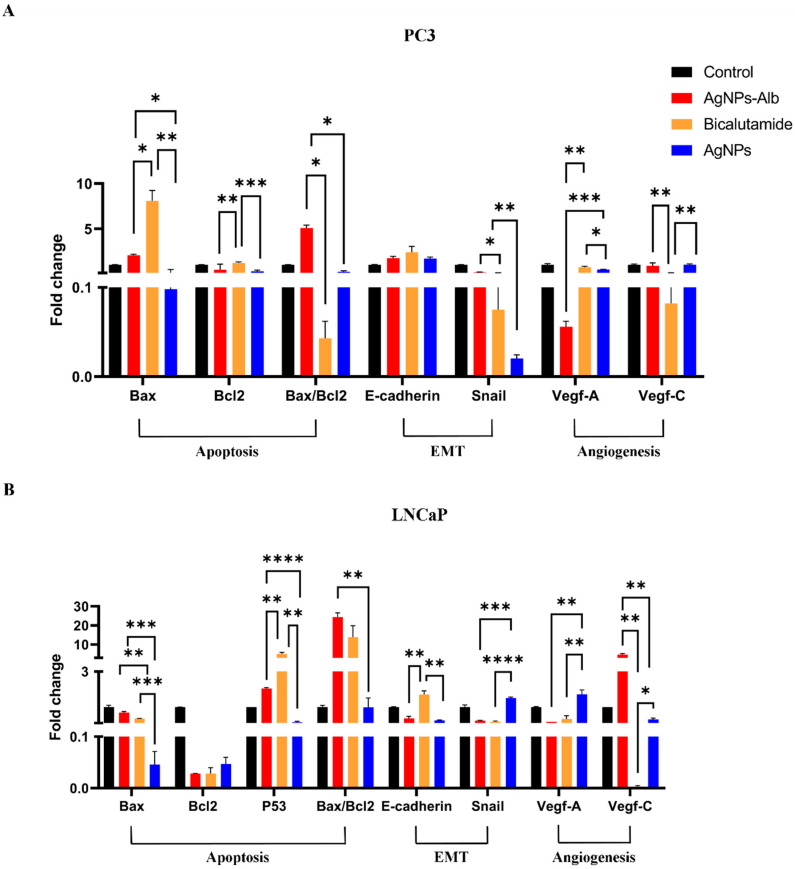
Analysis of gene expression in the PC3 (**A**) and LNCaP (**B**) prostate cancer cell lines treated with AgNPs, AgNPs-Alb, and Bicalutamide over 24 h. Results are presented as the mean ± SD from three separate experiments. Statistical significance thresholds were set at * *p* < 0.05, ** *p* < 0.01, *** *p* < 0.001, and **** *p* < 0.0001.

The PC3 and LNCaP prostate cancer cell lines were treated with AgNPs, AgNPs-Alb, and Bicalutamide for 24 h. Following this treatment, the expression levels of apoptosis-related genes (*Bax*, *Bcl2*, *P53*), angiogenesis and lymph angiogenesis genes (*VEGF-A*, *VEGF-C*), and genes associated with the EMT pathway (*Snail* and *E-cadherin*) were examined through Real-Time PCR (Figure 9).

In the PC3 cell line, the expression of Snail was significantly decreased in all treated groups (*p* < 0.0001). The treatment with AgNPs resulted in a greater reduction in Snail expression compared to AgNPs-Alb (*p* < 0.01), though the difference between AgNPs and Bicalutamide was not statistically significant. *E-cadherin* expression was significantly upregulated in all treated groups when compared to the control (*p* < 0.05), though the increase was similar across all treatment groups (*p* > 0.05). *VEGF-A* levels were significantly reduced in the AgNPs-Alb group compared to both AgNPs (*p* < 0.001) and Bicalutamide (*p* < 0.01). In contrast, the expression of *VEGF-C* was significantly reduced by Bicalutamide more than by any other treatments (*p* < 0.05). Regarding apoptosis-related genes, the *Bax/Bcl2* ratio was notably higher in the AgNPs-Alb group compared to both Bicalutamide (*p* < 0.05) and AgNPs (*p* < 0.05). Additionally, AgNPs treatment led to a higher increase in this ratio than Bicalutamide (*p* < 0.05).

In the LNCaP cell line, AgNPs-Alb significantly reduced Snail expression compared to AgNPs (*p* < 0.001). However, *E-cadherin* expression was similar between the AgNPs and AgNPs-Alb groups (*p* > 0.05). *VEGF-A* expression was significantly lower in the AgNPs-Alb group compared to both AgNPs and Bicalutamide (*p* < 0.01). On the other hand, AgNPs treatment resulted in a more pronounced reduction in *VEGF-C* expression than AgNPs-Alb (*p* < 0.01). The *Bax/Bcl2* ratio was significantly increased in the AgNPs-Alb group compared to the AgNPs group (*p* < 0.01).

In PC3 cells, the Bicalutamide treatment resulted in a greater reduction in mRNA levels for VEGF-C and an increased Bax/Bcl2 ratio. The AgNPs-Alb treatment led to a significant decrease in VEGF-A expression, while the AgNPs group showed a notable reduction in BCL2 levels. Meanwhile, in LNCaP cells, Bicalutamide treatment caused a greater decrease in mRNA levels for VEGF-C, Snail, and BCL2. Similarly, the AgNPs-Alb group demonstrated decreased expression of VEGF-A and BCL2, whereas the AgNPs treatment significantly downregulated P53. Notably, the AgNPs-Alb treatment showed the most prominent increase in the Bax/Bcl2 ratio in LNCaP cells, suggesting its potent pro-apoptotic effects.

## 4. Discussion

AgNPs show promising potential in cancer treatment by selectively interfering with the mitochondrial respiratory chain, which leads to the formation of reactive oxygen species (ROS) and blocks ATP production, ultimately causing DNA damage [25]. However, their use in therapy faces challenges such as quick opsonization and rapid clearance by the kidneys, resulting in a short time in the bloodstream and low concentrations at the targeted tissues [26,27,28]. On the other hand, albumin-based nanoparticles are highly effective for delivering therapeutic agents due to their ability to carry large amounts of drugs, their biodegradability, compatibility with biological systems, and their ability to bind to different ligands through covalent or non-covalent bonds [29]. To address these challenges, AgNPs-Alb have been developed to improve the delivery of AgNPs to prostate cancer cell lines like PC3 and LnCAP. The addition of albumin in AgNPs-Alb helps to maintain bioavailability and allows for targeted uptake by cancer cells through albumin receptor pathways [29].

The size of AgNPs-Alb nanoparticles, measured at 78.82 ± 15.61 nm using DLS, falls within the tumor microvessel size range of 70 to 1200 nm [30,31]. This size allows the nanoparticles to extravasate into tumor tissues while sparing normal tissues, effectively bypassing opsonization. AgNPs-Alb exhibit strong anti-proliferative effects against PC3 and LnCAP cell lines with dose-dependent toxicity. Notably, the IC50 value for AgNPs-Alb is lower than that of AgNPs alone, suggesting a more potent cytotoxic effect on cancer cells, likely due to increased uptake by these cells. This enhanced efficiency allows for reduced use of anticancer agents, minimizing potential damage to surrounding healthy tissues. AgNPs-Alb exhibit significantly greater anti-migratory effects on PC3 cells and more effectively inhibit colony formation in both PC3 and LnCAP cells compared to AgNPs. Additionally, AgNPs-Alb treatment results in a higher rate of total apoptosis in PC3 cells compared to AgNPs. Cell cycle analysis revealed an increase in the Sub-G1 phase in LnCAP cells following AgNPs-Alb treatment. Furthermore, AgNPs-Alb significantly suppresses mRNA expression of VEGF-A and elevates the Bax/Bcl2 ratio in both PC3 and LnCAP cells. An increase in P53 and E-cadherin expression, along with a reduction in VEGF-C expression, was also observed in LnCAP cells.

AgNPs-Alb induce a greater increase in ROS production within prostate cancer cells compared to AgNPs alone. This enhanced ROS generation is likely attributed to the higher cellular uptake of AgNPs and the amplified oxidative effect achieved by combining AgNPs with albumin. It is well-established that anticancer agents elevate ROS levels in treated cells, triggering pro-apoptotic signaling pathways that ultimately lead to apoptosis [32,33]. Furthermore, therapeutic strategies utilizing ROS-inducing agents have been proposed as effective approaches for selectively targeting and eliminating cancer cells [34]. Since AgNPs function as ROS-stressing agents in PC3 and LnCAP cell lines, AgNPs-Alb emerges as a promising chemotherapeutic candidate for the treatment of prostate cancer.

Our findings align with earlier research on the anticancer effects of silver nanoparticles and imply the activation of multiple mechanisms leading to cell death. De Matteis et al. suggested that the uptake of AgNPs by cells occurs via endocytosis, followed by their degradation in lysosomes. The resulting release of cytosolic silver ions may trigger elevated intracellular ROS production, leading to DNA damage and apoptosis mediated by mitochondrial pathways [35]. Similarly, Gurunathan et al. demonstrated that AgNPs exert cytotoxic effects in the breast cancer cell line MDA-MB-231 through a classic p53-dependent apoptotic pathway [36]. More recently, autophagy has been suggested as another potential mechanism. Lin et al. provided support for this hypothesis by showing that using an autophagy inhibitor enhances the anticancer effects of silver NPs [37]. Additionally, Yang et al. reported that silver NPs contribute to cytotoxicity by interfering with the HIF signaling pathway, further underscoring the multifaceted nature of their anticancer activity [38].

Tumor angiogenesis plays a crucial role in supplying oxygen and essential nutrients to support tumor growth, while also facilitating the metastasis of tumor cells to distant sites [39,40]. As a result, targeting tumor angiogenesis has become a key strategy in cancer therapy, leading to the development and clinical implementation of numerous anti-angiogenic drugs [41]. Interestingly, various NPs, including chitosan, silica, selenium, gold, and silver NPs, have demonstrated the ability to inhibit angiogenesis, offering potential as effective anti-angiogenic agents [42,43]. The mechanisms underlying the anti-angiogenic activity of these NPs are diverse, often involving direct interactions with molecular targets such as VEGF165 and bFGF, as well as the downregulation of VEGFR2 and suppression of FGFR1, Erk1/2, Akt, and VEGFR2 phosphorylation [42]. Furthermore, the study has shown that both the coating material and size of the nanoparticles can significantly influence their inhibitory effects, particularly in mechanisms requiring the internalization and interaction of the NPs with specific target molecules [44].

In a study conducted by Azizi et al., AgNPs-Alb were developed, and their anticancer properties were evaluated against the MDA-MB-231 human breast cancer cell line. The results revealed that cell death was primarily induced through apoptosis. The LD50 of AgNPs-Alb for MDA-MB-231 cells was determined to be 5 μM, which is significantly lower compared to 152 μM for normal white blood cells, indicating a 30-fold increase in toxicity towards cancer cells. In comparison, our study demonstrated an IC50 value of 32 μM for AgNPs-Alb against PC3 prostate cancer cells, which was notably higher than the value reported in the Azizi et al. study. These findings suggest that AgNPs-Alb holds promise as a potent chemotherapeutic agent [45]. In a separate study, the anticancer effects of copper nanoparticles (CuNPs) and albumin-coated copper nanoparticles (CuNPs-Alb) were compared, with CuNPs-Alb displaying enhanced efficacy in reducing cancer cell viability while exhibiting lower toxicity towards normal cells. Specifically, treatment with CuNPs-Alb significantly increased ROS production in the MDA-MB-231 cell line compared to untreated cells. This heightened ROS production, observed 24 h post-treatment, strongly indicates apoptosis induction by CuNPs-Alb [45]. Similarly, Akhtar et al. studied the anticancer efficacy of bovine serum albumin-coated silver nanoparticles (BSA@AgNPs) against colorectal (HCT-116) and HeLa cancer cell lines. Their findings revealed a reduction in cancer cell populations following treatment with BSA@AgNPs compared to untreated controls [46]. Furthermore, albumin-capped AgNPs exhibited anticancer effects against breast cancer (MCF-7) at 80 μg/mL, intestinal colon cancer (HCT-116) at 60 μg/mL, and bone cancer (osteosarcoma MG-63) at 80 μg/mL. In contrast, normal fibroblast cells (3T3) demonstrated a higher IC50 value of 140 μg/mL, showcasing the selective cytotoxicity of albumin-capped AgNPs towards cancer cells [47]. The findings from these recent studies are consistent with the results of our study, further supporting the potential of albumin-coated nanoparticles, including AgNPs-Alb, as promising candidates for selective cancer therapy. Collectively, these studies emphasize the enhanced anticancer efficacy of albumin-based nanoparticle formulations and their ability to selectively target cancer cells while sparing normal cells.

We recommend that future research initiatives focus on more precise and impactful areas of investigation. Evaluating stability in physiological condition is vital for ensuring the relevance and reliability of our findings. Our AgNPs were incubated in cell culture medium for three weeks, during which no precipitation or turbidity was detected. Additionally, all experiments were performed in triplicate, yielding consistent results. These results provide an indirect but favorable indication of AgNPs’ stability. However, direct stability evaluation of these nanoparticles is highly warranted in future research. Moreover, we only performed TEM analysis of AgNPs due to the financial and technical limitations. TEM can provide valuable structural information for AgNPs-Alb nanoparticles and should be considered in future investigations. Furthermore, exploring the effects of AgNPs-Alb on cancer stem cells should be prioritized, as these cells are widely recognized for their role in disease relapse and therapeutic resistance. Additionally, to strengthen and validate our findings, the utilization of advanced molecular techniques, such as RNA interference (RNAi) and virus-mediated gene therapy for gene knockdown and overexpression, could provide deeper insights into the underlying mechanisms of action. Investigations employing xenograft mouse models should also be considered to examine the in vivo adaptive responses of prostate cancer cells to AgNPs-Alb treatment, thereby offering translational relevance to clinical settings. Moreover, protein level analysis of critical factors such as VEGF-C, KLK3, and components of the EMT system could be performed using the Western blot technique to further elucidate AgNPs-Alb’s molecular impact in cancer progression and therapy.

## 5. Conclusions

This study is the first to demonstrate that AgNPs-Alb exhibits stronger anticancer and cytotoxic effects compared to AgNPs alone in prostate cancer cell lines, specifically by influencing migration, colony formation, apoptosis, cell cycle progression, and gene expression profiles. Importantly, the effects were more pronounced in PC3 cells compared to LnCAP cells, indicating a differential sensitivity and highlighting AgNPs-Alb’s potential as a targeted treatment for advanced prostate cancer.

## Figures and Tables

**Table 1:** The sequences of primers used for real-time PCR for seven target genes and *B2 M* as the housekeeping gene.

Gene	Forward Primer (5′-3′)	Reverse Primer (5′-3′)
***P53***	ACTGGGACGGAACAGCTTTG	TTGGGCAGTGCTCGCTTAG
***E-cadherin***	TCGTAACGACGTTGCACCAA	TTCGGAACCGCTTCCTTCAT
***SNAIL***	TAGCGAGTGGTTCTTCTGCG	AGGGCTGCTGGAAGGTAAAC
***VEGF-C***	GCTTCTTCTCTGTGGCGTGT	CTTTGCTTGCATAAGCCGTGG
***VEGF-A***	CTTCAAGCCATCCTGTGTGC	TGGCCTTGGTGAGGTTTGAT
***Bcl2***	CCCCGCGACTCCTGATTCAT	CAGTCTACTTCCTCTGTGATGTTGT
***Bax***	CGGGTTGTCGCCCTTTTCTAC	AGTCCAATGTCCAGCCCATGA
***B2M***	TGTCTTTCAGCAAGGACTGGT	TGCTTACATGTCTCGATCCCAC

**Table 2:** The IC_50_ values of prostate cancer cell lines after 24 h treatment with different agents. An unpaired Student’s *t*-test was performed to compare the treatment groups with the AgNPs group.

Cell Lines	Treatments		
	AgNPs	AgNPs-Alb	Bicalutamide
**LNCaP**	110 ± 1.74 μM	95 ± 1.32 μM (*p* < 0.001)	256 ± 0.88 μM (*p* < 0.0001)
**PC3**	48 ± 0.32 μM	32 ± 0.08 μM (*p* < 0.0001)	384 ± 4.36 μM (*p* < 0.0001)

## Data Availability

The original contributions presented in this study are included in the article. Further inquiries can be directed to the corresponding author(s).
